# The complete mitochondrial genome of Robin Accentor *Prunella rubeculoides* (Passeriformes: Prunellidea)

**DOI:** 10.1080/23802359.2020.1832596

**Published:** 2020-11-03

**Authors:** Shan Xia, Lu Qiao, Kong Yang, Bisong Yue, Nan Yang

**Affiliations:** a College of Chemistry and Life Sciences, Sichuan Provincial Key Laboratory for Development and Utilization of Characteristic Horticultural Biological Resources, Chengdu Normal University, Chengdu, China; b Sichuan Key Laboratory of Conservation Biology on Endangered Wildlife, College of Life Sciences, Sichuan University, Chengdu, China; c Institute of Qinghai-Tibetan Plateau, Southwest Minzu University, Chengdu, China

**Keywords:** *Prunella rubeculoides*, complete mitochondrial genome gene arrangement

## Abstract

In this paper, we determined and described the complete mitochondrial genome of Robin Accentor (*Prunella rubeculoides*), the first complete mitogenome. The complete genome of *P. rubeculoides* was 16,796 bp in length and contained 13 protein-coding genes, 22 transfer RNA genes, two ribosome RNA genes, and one non-coding control region. The overall base composition of the mitochondrial DNA was 29.7% for A, 23.7% for T, 15.6% for G, and 31.0% for C, with a GC content of 46.6%. This information of *P. rubeculoides* mitogenome is significance for phylogenetic studies of the family Prunellidea.

Robin Accentor (*Prunella rubeculoides*) is a grayish and medium-size bird (16 cm) within the family Prunellidea. Its chest is chestnut brown, while its head, throat, upper body, wings, and tail are smoke-brown. Its upper back has a faint black stripe. It has a narrow black collar between its gray throat and chestnut-brown chest. The rest of its body is white (Birdnet 2014). The bird distributes in Bhutan, China, India, Nepal, and Pakistan, which is completely endemic to the Palearctic (Surhone et al 2011). The species was rated in the least concerned species and placed on the IUCN's 2016 Red List of Least Concern Species (LC) (IUCN 2016). In China, it is found in the Himalayas, central China and south of the Qinghai-Tibet Plateau (Surhone et al. 2011). It is an uncommon resident bird, found in the meadows and rhododendron and willow thicles of northern and eastern Qinghai, western Gansu and Sichuan, and southern Tibet, 3600–4900 m above sea level (Birdnet 2014). However, there is no molecular data reported on *P. rubeculoides*. Therefore, in order to better understand the phylogeny and evolution of this species or subspecies in the Prunellidea family, we sequenced the complete mitochondrial genome of *P. rubeculoides.*

A natural dead *P. rubeculoides* was collected from Xiejia village, Marong township, Baiyu county, Ganzi Tibetan Autonomous region, Sichuan province. The specimens is now in the Key Laboratory of Bioresources and Ecoenvironment, Sichuan University. The stored number of the sample is 2019PD-1. Total genomic DNA was extracted from muscle tissue using standard phenol-chloroform methods (Sambrook and Russell 2001). The complete mitochondrial genome was amplified using PCR and PCR produces were sequenced by ABI PRISM 3730 DNA sequencer. The complete mitochondrial genome of *P. rubeculoides* was submitted to the NCBI database under the accession number QYL.sqn DG2 MT903225. The full genome of *P. rubeculoides* mitochondrial is a circular DNA molecule and is 16,796 bp in length. The overall base composition was 29.7% A, 23.7% T, 15.6% C, and 31.0% G, with a GC ratio of 46.6%. The complete mitogenome of *P. rubeculoides* included 13 protein-coding genes, two ribosomal RNA genes (12S rRNA and 16S rRNA), 22 transfer RNA (tRNA) genes, and one non-coding control region (D-loop). Among these genes, ND6 and eight tRNA genes (tRNA-Gln, tRNA-Ala, tRNA-Asn, tRNA-Cys, tRNA-Tyr, tRNA-Ser, tRNA-Glu, and tRNA-Pro) located on the light strand (L-strand), whereas other genes located on the heavy strand (H-strand). The 12S and 16S rRNA genes located between tRNA-Phe and tRNA-Leu contains 974 and 1596 bp, respectively.

Eleven of the 13 PCGs used ATG as the start codon, while COX1 and ND6 selected GTG and CTA, respectively. The PCGs have six types of termination codon, including TAA for ATP8, ND3, ND4 and Cytb, TAG for ATP6, AGA for ND1 and ND5, AGG for COX1, ‘T––’ for COX2, COX3, ND4 and ND6, and ‘TA–’ for ND2. Some birds have been found to have single insertion mutations in ND3, but the *P. rubeculoides* has WOC (without extra Cytosine) ND3 (Yan et al. 2017). Unlike these mitochondrial genomes of many other bird, which have a pseudo—control region (Song et al. 2015), the mitochondrial genome of *P. rubeculoides* has only one single control region, and no repeat sequence was found in the control region.

A neighbor-joining (NJ) tree of 11 species from Passeriformes was constructed based on the dataset of 13 concatenated mitochondrial PCGs using MEGA 7 (MEGA Inc., Englewood, NJ) with 1000 bootstrap replicates. The reconstructed phylogenetic tree supports the position of *P. rubeculoides* in the Prunellidea (Figure 1) with high bootstrap values. As can be seen from the tree (Figure 1), the results are similar to previous studies on *Prunella montanella* in the same genus (Yao et al. 2015). The sequence data will be useful for the phylogenetic studies of the family Prunellidea species in the future.

**Figure 1.**
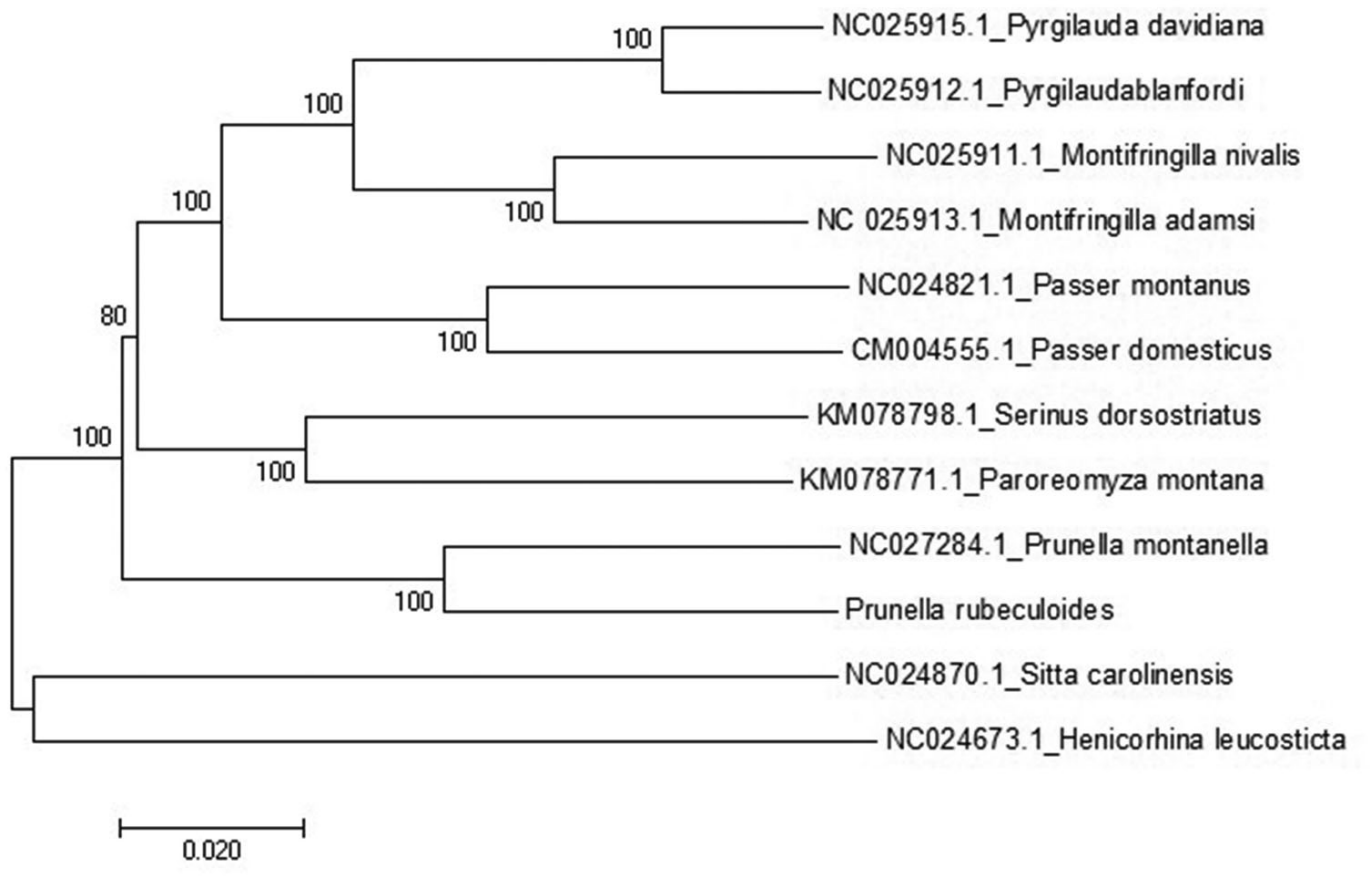
Phylogenetic tree of neighbor-joining (NJ) method based on the 13 mitochondrial PCGs nucleotide sequences of 11 species from Passeriformes published.

## Data Availability

We also confirm that the data supporting the findings of this study are available within the article and in Genbank. https://www.ncbi.nlm.nih.gov/Genbank/update.html
